# Prognostic significance and clinicopathological associations of tumor budding and poorly differentiated clusters in endometrioid endometrial carcinoma

**DOI:** 10.55730/1300-0144.6188

**Published:** 2026-01-29

**Authors:** Hanife Seda MAVİLİ, Ömer ATMIŞ, Fatma Seher PEHLİVAN, Fatma Sezen ÇAVDAROĞLU DEVECİ, Ali Rıza KANDİLOĞLU

**Affiliations:** Department of Pathology, Faculty of Medicine, Manisa Celal Bayar University, Manisa, Turkiye

**Keywords:** Tumor budding, poorly differentiated clusters, MELF pattern, endometrioid endometrial carcinoma

## Abstract

**Background/aim:**

Tumor budding (TB) and poorly differentiated clusters (PDCs) are histopathological parameters that have been associated with poor prognosis in various malignancies, particularly colorectal carcinoma. Although numerous studies have demonstrated an association between the microcystic, elongated, and fragmented (MELF) pattern in endometrial carcinomas and lymphovascular invasion as well as lymph node metastasis, the literature regarding TB and PDCs in this context remains limited.

This study aimed to investigate the potential associations of the MELF pattern, TB, and PDCs with overall survival, progression-free survival, and clinicopathological parameters in cases of endometrioid endometrial carcinoma (EEC).

**Materials and methods:**

A total of 190 cases diagnosed with EEC through hysterectomy specimens between 2010 and 2023, with complete hospital records, were selected from the archives of the Department of Pathology at Manisa Celal Bayar University Faculty of Medicine.

**Results:**

The presence of TB and PDCs was significantly associated with high histological grade (p < 0.001), deep myometrial invasion (p < 0.001), lymphovascular invasion (p < 0.001), lymph node metastasis (p < 0.001), cervical stromal involvement (p < 0.001), serosal involvement (p < 0.001), advanced stage (p < 0.001), and larger tumor size (p = 0.042 and p = 0.027, respectively). The presence of TB and PDCs was found to be associated with reduced overall survival (p = 0.001 and p < 0.001, respectively) and reduced progression-free survival (p = 0.043 and p = 0.004, respectively). The MELF pattern was not significantly associated with overall survival (p = 0.772).

**Conclusion:**

These findings suggest that the presence of TB and PDCs may be valuable in stratifying prognostic risk in EEC and support the inclusion of these parameters in routine pathology reporting.

## 1. Introduction

Endometrial carcinoma is the most common malignancy of the female genital tract [1]. The most frequently encountered subtype (75%–80%) is endometrioid endometrial carcinoma (EEC) [2]. Depth of myometrial invasion is an important parameter incorporated into the International Federation of Gynecology and Obstetrics (FIGO) staging system for endometrial carcinoma. Additionally, established prognostic factors for tumor recurrence include histological grade, patient age, tumor size (>2 cm), lymphovascular invasion (LVI), and metastasis to pelvic and paraaortic lymph nodes [3].

Recently, patterns of myometrial invasion, particularly in low-stage EEC, have been emphasized as potential prognostic factors [4]. Numerous studies have demonstrated that the microcystic, elongated, and fragmented (MELF) pattern is associated with lymph node metastasis (LNM) and deep myometrial invasion; however, its independent prognostic value remains uncertain [5–7].

The standardized assessment of tumor budding (TB) in colorectal carcinomas is performed according to the recommendations of the 2016 International Tumor Budding Consensus Conference [8]. Tumor budding has also been identified as an important prognostic factor in various carcinomas [9]. In the limited available studies, TB in EEC has been shown to be associated with adverse clinicopathological parameters, including LVI, LNM, deep myometrial invasion, and reduced overall survival [10–17]. However, there is no standardized assessment of TB in endometrial carcinomas.

Poorly differentiated clusters (PDCs) have also been shown to represent adverse prognostic factors in colorectal carcinomas [18]. A study evaluating PDCs in EEC demonstrated that PDCs presence was associated with reduced progression-free and overall survival [13].

In recent years, TB and PDCs have been investigated as prognostic markers in EEC, consistent with their established roles in other malignancies, including colorectal carcinomas. In the present study, the prognostic value of TB and PDCs and their associations with other clinicopathological parameters in EEC were investigated, with the aim of contributing to improved risk stratification and treatment planning.

## 2. Materials and methods

### 2.1. Patients and specimens

A total of 190 cases diagnosed with EEC in hysterectomy specimens between 2010 and 2023 were selected from the archives of the Department of Pathology, Faculty of Medicine, Manisa Celal Bayar University. Only cases with a diagnosis of endometrioid carcinoma that underwent surgical staging via hysterectomy were included in the study. Carcinomas confined to the endometrium or polyps without myometrial invasion were excluded. Patients who had received preoperative chemotherapy, radiotherapy, or hormonal therapy, or who had undergone incomplete surgical procedures, were also excluded. The study cohort consisted of 190 patients with complete follow-up, all of whom were included in the survival analyses. The study was approved by the Health Sciences Ethics Committee of Manisa Celal Bayar University Faculty of Medicine (Approval no: 20.478.486/2472; 5 June 2024).

Hematoxylin and eosin (H&E)–stained slides were reevaluated, and the diagnoses were confirmed according to the 2020 World Health Organization classification [2]. Hospital records were reviewed to determine disease stage, the presence of distant metastases based on radiologic or clinical findings, and recurrence status. Patients’ age and survival status were recorded. Histopathological parameters, including tumor grade based on the FIGO system [19], were reassessed during slide review.

The pT stages were noted according to the 8th edition of the American Joint Committee on Cancer (AJCC) staging system. Staging was performed following the 2023 FIGO surgical staging criteria [20]. Stages I and II were grouped as early stage, whereas stages III and IV were classified as advanced stage. Due to the limited number of cases in pT2 and higher categories, pT1 and pT2 cases were combined into a single group, whereas pT3 and pT4 cases were analyzed as a separate group.

In the survival analysis, overall survival (OS) was defined as the time from the date of diagnosis to death from any cause or censoring at the last follow-up date (1 June 2024). Progression-free survival (PFS) was defined as the time from diagnosis to the first documented disease progression or recurrence. Disease progression and/or recurrence were ascertained based on radiologic imaging findings, histopathologic confirmation when available, and review of clinical follow-up records documented in the hospital information system. For PFS analysis, deaths occurring in the absence of documented progression or recurrence were censored at the date of death and were not considered PFS events. Patients without documented progression, recurrence, or death were censored at the date of the last clinical follow-up.

### 2.2. Histopathological evaluation

All H&E–stained slides containing tumor tissue were retrieved from the archive and reexamined under a standard light microscope to evaluate TB and PDCs.

**Figure 1:**
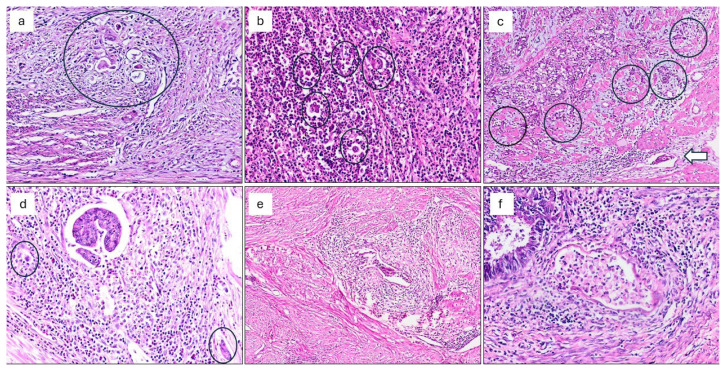
(a) Tumor budding (TB) and poorly differentiated clusters (PDCs) (circles) (H&E stain, original magnification ×200). (b) Numerous tumor buds and PDCs (circles) (H&E stain, original magnification ×200). (c) Poorly differentiated clusters and TB (circles) accompanied by lymphovascular invasion (arrow) (H&E stain, original magnification ×100). (d) Microcystic, elongated, and fragmented (MELF) pattern (center), TB (circle, left), and PDCs (circle, right) (H&E stain, original magnification ×200). (e, f) MELF pattern (e: H&E stain, original magnification ×100; f: H&E stain, original magnification ×200). Abbreviations: TB, tumor budding; PDCs, poorly differentiated clusters; MELF, microcystic, elongated, and fragmented pattern.

Sections representing the deepest extent of tumor invasion and the invasive front with the highest density of TB and PDCs were selected for analysis. Tumor budding (TB) was defined as isolated single tumor cells or small clusters composed of fewer than five tumor cells at the invasive front (Figures 1a–1d) [8].

There is no standardized guideline for evaluating TB in EEC. Different cut-off values for TB positivity in EEC have been reported in the literature. Although the presence of five or more TB foci is most commonly considered positive [11,14,15], some studies have considered even a single TB focus to be positive [12,13]. Additionally, publications categorizing TB into low- and high-grade groups have used cut-off values of five or more TB foci [10] or three or more TB foci to define high grade [16].

In the study by Stögbauer et al., various methodologies for identifying TB (e.g., TB in one high-power field [HPF; ×40] or TB in 10 HPFs) were suggested. Although univariate analyses yielded significant results across different methodologies, assessment of TB in one HPF demonstrated superior performance in multivariable analysis. Accordingly, assessment of TB in one HPF was recommended for clinical practice [16].

All evaluations were performed on conventional glass slides using a standard light microscope (Nikon Eclipse E600; Nikon Corporation, Tokyo, Japan) with a ×20 objective lens. For each case, all available tumor-containing slides were reviewed, and the slide showing the highest density of TB and PDCs at the invasive front was selected for scoring. The evaluation focused on the area demonstrating the highest density of TB and PDCs (hot spot). When multiple potential hot spot areas were identified, the field with the highest number of TB and PDCs was selected for analysis. TB and PDCs counts were performed in a single high-power field (HPF) at ×20 magnification, corresponding to an area of approximately 0.785 mm^2^. TB was defined as isolated single tumor cells or clusters composed of fewer than five tumor cells, whereas PDCs was defined as nongland-forming tumor cell clusters composed of five or more tumor cells at the invasive margin (Figures 1a–1d) [21]. All assessments were carried out on glass slides.

Cut-off values for TB and PDCs were predefined based on previously published studies in EEC [12,13,16]. Although a threshold of five or more buds or clusters has frequently been used in the literature, the limited number of cases meeting this criterion in the present cohort precluded reliable statistical analysis. Review of the distribution of TB and PDCs counts in the present cohort demonstrated that cut-off values of one or more and three or more allowed discrimination between mere presence and increased burden while preserving statistical robustness. Accordingly, these thresholds were applied a priori and were not derived through post hoc analysis.

The presence of the MELF pattern was defined as invasion characterized by microcystic, elongated, and/or slit-like glands composed of squamoid or eosinophilic tumor cells, frequently exhibiting intraluminal fragmentation and neutrophilic infiltration (Figures 1d–1f) [22].

Histopathological evaluation of TB, PDCs, and the MELF pattern was performed independently by three experienced pathologists (H.S.M., A.R.K., and Ö.A.). All observers were blinded to clinical, pathological, and survival-related data at the time of assessment. All cases were reviewed using predefined diagnostic criteria. In cases of disagreement, consensus was achieved through simultaneous review using a multiheaded microscope.

### 2.3. Assessment of mismatch repair status

Mismatch repair (MMR) status was evaluated by immunohistochemistry (IHC) using antibodies against the four core MMR proteins—MLH1 (clone M1), PMS2 (clone A16–4), MSH2 (clone G219–1129), and MSH6 (clone SP93)—on representative formalin-fixed, paraffin-embedded tumor sections. Tumors demonstrating complete loss of nuclear expression of at least one MMR protein in tumor cells, with preserved internal control staining in stromal and inflammatory cells, were classified as MMR-deficient (MMRd). Tumors with intact nuclear expression of all four MMR proteins were classified as MMR-proficient (MMRp). Cases with technically inadequate or unavailable staining were categorized as having an unknown MMR status.

### 2.4. Statistical analysis

All data obtained from the study were analyzed using SPSS version 25 (IBM Corp., Armonk, NY, USA). The Kaplan–Meier method was used to generate survival curves, and the log-rank test was employed for comparison of survival distributions. For survival analysis, univariate Cox proportional hazards regression models were constructed to estimate hazard ratios (HRs) and 95% confidence intervals (CIs). Variables found to be significant in univariate analyses were subsequently included in multivariable Cox proportional hazards regression and binary logistic regression models. The chi-square test and Fisher’s exact test were used to assess associations between categorical nonsurvival variables. Spearman’s rank correlation coefficient was used to assess correlations between continuous or ordinal variables. Results were considered statistically significant at p < 0.05.

## 3. Results

### 3.1. Clinicopathological characteristics of the study cohort

The age and clinicopathological characteristics of the 190 patients with EEC included in the study are presented in Table 1. During the follow-up period, 36 patients died, and disease progression and/or recurrence was observed in eight patients. The number of cases with five or more TB foci was 13, those with five or more PDCs were three, and only one case demonstrated 10 or more TB foci. No cases demonstrated 10 or more PDCs. Due to the limited number of cases demonstrating five or more or 10 or more TB foci or PDCs, cut-off values of one and three were adopted instead of the tripartite classification used in colorectal carcinoma.

### 3.2. Associations of tumor budding, poorly differentiated clusters, and the MELF pattern with clinicopathological parameters

The associations of TB and PDCs with clinicopathological parameters according to different cut-off values are presented in Table 2. The presence of TB, based on cut-off values of one and three, was significantly associated with high histological grade, deep myometrial invasion, LVI, LNM, cervical stromal involvement, serosal involvement, advanced stage (p < 0.001), and larger tumor size (p = 0.042 and p = 0.019, respectively). A significant association was observed between TB and distant metastasis at a cut-off value of one (p < 0.001), whereas no statistically significant association was observed at a cut-off value of three (p = 0.087).

The association of PDCs presence at a cut-off value of one with clinicopathological parameters was similar to that observed for TB at the same threshold. However, at a cut-off value of three, no statistically significant associations were observed with cervical stromal involvement, larger tumor size, or distant metastasis (p = 0.224, p = 0.313, and p = 0.192, respectively). In MMRp tumors, the MELF pattern, TB, and PDCs (each at a cut-off value of one) were observed significantly less frequently compared with MMRd tumors (p = 0.011, p = 0.006, and p = 0.016, respectively). At a cut-off value of one, both TB and PDCs presence demonstrated stronger associations with multiple clinicopathological parameters.

The associations of the MELF pattern with clinicopathological parameters resembled those observed for TB at a cut-off value of one. However, no statistically significant associations were observed with histological grade or distant metastasis (p = 0.102 and p = 0.809, respectively).

When low-grade (grade I–II), grade I, stage I, and pT1 tumors were evaluated separately, the presence of the MELF pattern, TB, and PDCs (cut-off value of one) was significantly associated with LVI (p < 0.001). A similar association was observed at a cut-off value of three in low-grade (p < 0.001) and grade I tumors for both TB and PDCs (p < 0.001 and p = 0.004, respectively). However, in stage I and pT1 cases, this association was observed only for TB (p < 0.001) and not for PDCs (p = 0.104 and p = 0.102, respectively). In MMRd cases, both TB (cut-off values of one and three; p < 0.001) and PDCs presence (cut-off value of one: p < 0.001; cut-off value of three: p = 0.009) were significantly associated with LVI, whereas MELF pattern presence did not reach statistical significance (p = 0.051).

In low-grade and grade I cases, the presence of the MELF pattern, TB, and PDCs (cut-off value of one) was significantly associated with LNM (p < 0.001). This association remained statistically significant in low-grade cases at a cut-off value of three (p < 0.001). However, in high-grade cases, no statistically significant associations was observed between the MELF pattern, TB, or PDCs presence and LNM (p = 0.541, p = 0.308, and p = 0.308, respectively).

In MMRd cases, the MELF pattern (p = 0.004), TB (cut-off value of one: p = 0.001; cut-off value of three: p = 0.031), and PDCs presence (cut-off value of one: p = 0.006; cut-off value of three: p = 0.018) were significantly associated with LNM.

The associations of these three parameters with LNM in stage I cases were evaluated together with stage II cases due to the absence of LNM events in stage I. In early-stage cases, a TB count of three or higher was significantly associated with LNM (p = 0.001), whereas the MELF pattern did not reach statistical significance (p = 0.065).

In cases with less than 50% myometrial invasion, the MELF pattern, TB (cut-off values of one and three; p < 0.001), and PDCs presence (cut-off value of one: p < 0.001; cut-off value of three: p = 0.009) were significantly associated with LVI.

### 3.3. Associations between clinicopathological parameters and survival outcomes

Overall survival decreased with increasing histological grade and disease stage (p < 0.001 for both). Additionally, deep myometrial invasion, cervical stromal involvement, serosal involvement, LVI, LNM, and distant metastasis were significantly associated with reduced overall survival (p = 0.030, p = 0.031, p < 0.001, p < 0.001, p = 0.010, and p < 0.001, respectively).

When cases were stratified according to the pattern of myometrial invasion, the pushing pattern was associated with the most favorable prognosis, the infiltrative pattern with the least favorable prognosis, and the MELF and adenomyosis-like patterns with an intermediate prognosis (p = 0.006).

A tumor size of 4 cm or greater was not significantly associated with overall survival (p = 0.062). Higher histological grade and the presence of distant metastasis were significantly associated with reduced progression-free survival (p = 0.019 and p < 0.001, respectively).

### 3.4. Associations of tumor budding, poorly differentiated clusters, and the MELF pattern with survival outcomes

**Figure 2:**
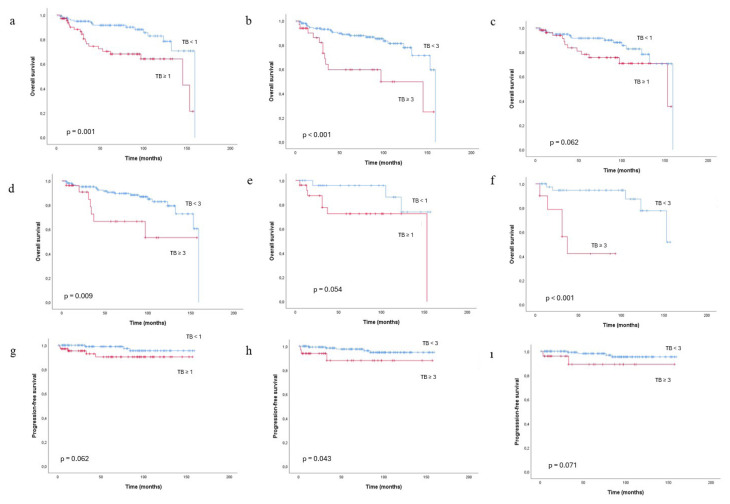
(a–ı) Overall and progression-free survival analyses using the Kaplan–Meier method. Tumor budding (TB) represents a significant or marginal prognostic parameter for overall survival in the overall cohort (a,b), low-grade cases (c,d), and mismatch repair–deficient cases (MMRd) (e,f); and for progression-free survival in the overall cohort (g,h) and low-grade cases (ı).

**Figure 3:**
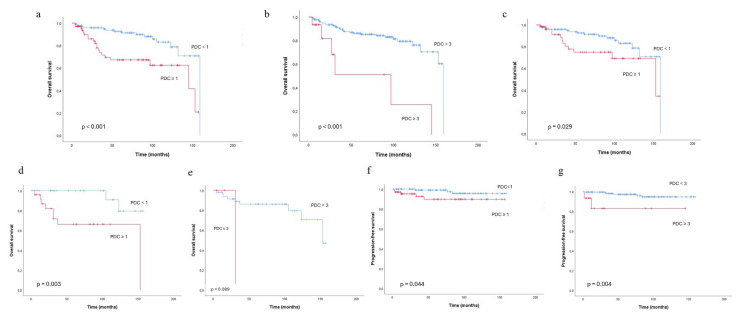
(a–g) Overall and progression-free survival analyses using the Kaplan–Meier method. Poorly differentiated clusters (PDCs) represent significant or marginal prognostic parameters for overall survival in the overall cohort (a,b), low-grade cases (c), and mismatch repair–deficient (MMRd) cases (d,e); and for progression-free survival in the overall cohort (f,g).

The associations of TB and PDCs with overall and progression-free survival according to different cut-off values are presented in Figures 2a–2ı and 3a–3g. The median follow-up time was 68 months, as estimated by the Kaplan–Meier method. During the follow-up period, 36 patients (18.9%) died. TB was significantly associated with reduced overall survival in both Kaplan–Meier and Cox proportional hazards regression analyses. Patients with TB of one or more demonstrated significantly worse overall survival compared with TB-negative cases (log-rank p = 0.001) (Figure 2a), with an approximately threefold increased risk of death (HR = 2.94, 95% CI: 1.50–5.76; p = 0.002). This prognostic effect was more pronounced in patients with a higher TB burden (Figure 2b); TB of three or more was associated with a further increase in mortality risk (HR = 3.47, 95% CI: 1.72–7.00; p = 0.001). Similarly, the presence of PDCs was strongly associated with reduced overall survival (Figures 3a and 3b). Patients with PDCs of one or more had significantly worse outcomes than PDCs-negative cases (log-rank p < 0.001), with a more than threefold increased risk of death (HR = 3.24, 95% CI: 1.66–6.32; p = 0.001). Notably, high-burden PDCs (three or more) identified a subgroup with particularly poor prognosis, demonstrating an almost fivefold increased risk of death compared with patients with lower PDCs counts (fewer than three) (HR = 4.98, 95% CI: 2.03–12.17; p < 0.001) (Table S1). Overall, both TB and PDCs demonstrated a clear burden-dependent adverse effect on overall survival, supporting their role as strong prognostic indicators in EEC. Kaplan–Meier analyses demonstrated progressively worse survival with increasing TB and PDCs categories (0, 1–2, and ≥3; log-rank p < 0.001 for both; patient and event numbers are summarized in Table S2). Consistently, Cox proportional hazards regression analysis confirmed that each incremental increase in TB and PDCs burden was associated with a significantly higher risk of death (TB: HR = 2.05, 95% CI: 1.40–3.00; PDCs: HR = 2.66, 95% CI: 1.68–4.21; both p < 0.001). However, the presence of the MELF pattern was not significantly associated with overall survival (log-rank p = 0.772).

In low-grade tumors (Figures 2c and 2d), a TB count of three or more was significantly associated with reduced overall survival in both Kaplan–Meier and Cox proportional hazards regression analyses (n = 25, events = 7 versus n = 149, events = 21; log-rank p = 0.009; HR = 3.00, 95% CI: 1.26–7.14; p = 0.013), whereas a TB count of one or more did not reach statistical significance (log-rank p = 0.062). Similarly, the presence of PDCs of one or more was associated with worse overall survival in low-grade tumors (n = 52, events = 12 versus n = 122, events = 16), as demonstrated by Kaplan–Meier analysis (log-rank p = 0.029) (Figure 3c). This association remained significant in Cox proportional hazards regression analysis, with PDCs positivity conferring a more than twofold increased risk of death (HR = 2.28, 95% CI: 1.07–4.86; p = 0.034) (Table S3).

In MMRd tumors (Figures 2e and 2f), a TB count of three or more was significantly associated with reduced overall survival in both Kaplan–Meier and Cox proportional hazards regression analyses (three or more buds: n = 10, events = 5; fewer than three buds: n = 43, events = 5; log-rank p < 0.001; HR = 12.28, 95% CI: 2.39–63.09; p = 0.003). A TB count of one or more did not reach statistical significance (log-rank p = 0.054). Similarly, the presence of PDCs of one or more was significantly associated with reduced overall survival in both Kaplan–Meier (Figure 3d) and Cox proportional hazards regression analyses (PDCs of one or more: n = 25, events = 8; PDCs fewer than one: n = 28, events = 2; log-rank p = 0.003; HR = 8.51, 95% CI: 1.67–43.29; p = 0.010).

However, in high-grade tumors, no statistically significant associations were observed between the presence of TB or PDCs and overall survival.

Kaplan–Meier analysis demonstrated significantly worse PFS in patients with a TB count of three or more (log-rank p = 0.043) (Figure 2h), whereas Cox proportional hazards regression analysis demonstrated a strong but not statistically significant association between high TB burden and increased risk of progression (HR = 3.96, 95% CI: 0.94–16.63; p = 0.061). In Cox proportional hazards regression analysis for PFS, high PDCs burden (PDCs ≥ 3) was significantly associated with an increased risk of progression (HR = 7.66, 95% CI: 1.49–39.27; p = 0.015). Consistent with this finding, Kaplan–Meier analysis demonstrated significantly worse PFS in patients with a PDCs count of three or more compared with those with fewer than three PDCs (log-rank p = 0.004). For PFS, the presence of one or more PDCs was significantly associated with worse PFS on Kaplan–Meier analysis (log-rank p = 0.044) (Figures 3f and 3g). In Cox proportional hazards regression analysis, the presence of one or more PDCs was associated with an increased risk of progression; however, this association did not reach statistical significance (HR = 3.92; p = 0.062), likely reflecting the limited number of progression events. A TB count of one or more was not significantly associated with PFS in Kaplan–Meier analyses (p = 0.062). Overall, these findings suggest that high PDCs burden may represent a stronger and more robust predictor of disease progression than TB, particularly at higher cut-off thresholds. No statistically significant association was observed between the MELF pattern and progression-free survival (log-rank p = 0.663).

In low-grade cases, a TB count of three or more was not significantly associated with reduced progression-free survival (log-rank p = 0.071) (Figure 2ı), and no statistically significant association was observed with the MELF pattern (p = 0.346).

The only statistically significant association between the MELF pattern and survival was observed in cases with less than 50% myometrial invasion, in which the MELF pattern was associated with reduced progression-free survival (log-rank p = 0.003).

### 3.5. Multivariable analysis

A total of two multivariable models were constructed to evaluate the parameters associated with overall survival following univariate analysis. In the model excluding parameters contributing to stage determination (deep myometrial invasion, cervical stromal involvement, serosal involvement, and lymph node metastasis), only disease stage was identified as an independent prognostic factor for overall survival (p = 0.017). Conversely, in the second model, which included LVI, TB, and PDCs together with stage-related parameters, LVI and serosal involvement were identified as independent prognostic factors for overall survival (p = 0.010 and p = 0.009, respectively).

In the reduced multivariable logistic regression model for LNM (Table 3), TB (TB ≥ 1) and the presence of the MELF pattern remained independently associated with LNM (TB: OR = 11.14, 95% CI: 1.43–86.44; p = 0.021; MELF: OR = 3.26, 95% CI: 1.15–9.28; p = 0.027), whereas PDCs (PDCs ≥ 1) did not retain independent significance (p = 0.342). These results suggest that TB and the MELF pattern provide complementary information regarding the risk of LNM in EEC.

In multivariable analyses for LVI, TB (TB ≥ 1) and PDCs (PDCs ≥ 1) remained independently associated with LVI when evaluated in separate reduced models (TB: OR = 28.7, 95% CI: 11.1–74.3; p < 0.001; PDCs: OR = 31.3, 95% CI: 11.9–82.4; p < 0.001), whereas the MELF pattern did not demonstrate an independent association. Taken together, these findings suggest that TB and PDCs are closely linked to lymphovascular dissemination and may reflect aggressive tumor behavior beyond conventional histopathological patterns.

### 3.6. Association between tumor budding, poorly differentiated clusters, and the MELF pattern

The MELF pattern demonstrated a positive correlation with the number of TBs and PDCs. However, among the 58 cases exhibiting the MELF pattern, 40 cases had TB and PDCs at a cut-off value of one, while 22 cases had TB and seven had PDCs at a cut-off value of three.

Notably, at a cut-off value of one, the differences between the MELF pattern and the presence of TB or PDCs were minimal. Although no statistically significant association was observed between the MELF pattern and overall or progression-free survival, the presence of TB and PDCs, which were positively correlated with the MELF pattern, was significantly associated with reduced overall and progression-free survival.

## 4. Discussion

The majority of endometrial cancers consist of low-grade, early-stage endometrioid carcinomas, which generally have a favorable prognosis [23]. Although tumor staging remains essential for determining appropriate treatment and estimating patient prognosis, additional histological parameters, such as the pattern of myometrial invasion and TB, have recently been proposed as important prognostic factors [4]. In this study, given the limited prognostic value of the MELF pattern when considered alone, TB and PDCs were evaluated as easily recognizable morphological markers of adverse prognosis.

The first study to report the prognostic significance of TB in endometrial carcinomas was conducted in 2012 by Koyuncuoglu et al. [10]. They demonstrated that TB is an independent prognostic factor for survival and is associated with advanced stage and deep myometrial invasion [10]. In subsequent years, other studies have also supported their findings [11,12,15]. Furthermore, in contrast to Park et al. [11], Ocal and Guzelis, consistent with our findings, reported that TB is associated with cervical involvement [15].

Poorly differentiated clusters have been reported as adverse prognostic factors in colorectal cancer [18,21]; however, their role in EEC has not been extensively investigated. To date, the only study addressing this issue was conducted by Yamamoto et al. [13]. In the present study, similar to the findings of Yamamoto et al., both TB and PDCs were significantly associated with advanced stage, LNM, higher FIGO grade, LVI, and cervical involvement. Additionally, significant associations were identified with larger tumor size, serosal involvement, distant metastasis, and deep myometrial invasion.

Qi et al. reported that the presence of the MELF pattern and TB was associated with deep myometrial invasion, LVI, and LNM [14]. Our study confirms these findings as well. However, differences in cut-off values—such as one [12,13], three [16], and five [10,11,14,15]—have been reported across studies. In the present study, similar significant associations were observed between TB and PDCs and clinicopathological parameters at cut-off values of one and three; however, the cut-off value of one was associated with a greater number of parameters.

In terms of survival outcomes, stronger associations were identified for TB at a cut-off value of three and for PDCs at a cut-off value of one. For these reasons, the use of cut-off values of one and three may be considered in clinical practice.

Previous studies and risk scoring systems developed to stratify the risk of LNM in patients with EEC have incorporated various clinicopathological parameters [24,25]. However, debate persists regarding their clinical utility, particularly in the surgical management of early-stage EEC. Nevertheless, reliable predictors of LNM are required to optimize surgical and radiotherapeutic management.

As reported by Park et al. [11], and consistent with our findings, the presence of TB and PDCs were identified as independent predictors of LVI in multivariable analysis. In univariate analysis, both parameters were significant histological predictors of LNM. Additionally, multivariable analysis demonstrated that TB and the MELF pattern were independently associated with LNM.

The results also demonstrated that in low-grade cases, including grade I cases analyzed separately, the presence of the MELF pattern, TB, and PDCs at a cut-off value of one was significantly associated with LNM (p < 0.001). In early-stage cases, TB of three or more was significantly associated with an increased likelihood of LNM (p = 0.001). Similarly, Park et al., in their subgroup analysis of stage I EEC, reported a marginal association between TB and LNM as well as disease progression [11].

These findings, in line with the existing literature, suggest that TB and PDCs, together with the MELF pattern, may serve as useful risk parameters for clinical decision-making in EEC. In particular, these features may help inform decisions regarding pelvic and/or paraaortic lymphadenectomy. Such an assessment may also facilitate the identification of high-risk patients. From a practical perspective, these findings support the potential utility of incorporating TB and PDCs assessment into routine histopathological evaluation of EEC, particularly when interpreted alongside established parameters such as tumor grade, stage, and the MELF pattern. Given their association with LVI and LNM, TB and PDCs may provide additional risk stratification in early-stage disease, in which the extent of surgical staging remains controversial. Nevertheless, due to the retrospective design of the present study and the absence of standardized scoring systems for TB and PDCs in EEC, these parameters should currently be regarded as complementary prognostic markers rather than definitive indicators for lymphadenectomy.

Park et al. also reported the presence of TB within infiltrative and MELF patterns when evaluating patterns of myometrial invasion in conjuction with TB [11]. Similarly, in the present study, the vast majority of cases exhibiting TB and PDCs demonstrated infiltrative or MELF patterns. Park et al. identified a weak association between TB and overall survival, as well as a marginal association with disease progression [11]. However, Ocal and Guzelis reported that TB was associated with reduced overall survival [15], and Qi et al. reported associations with both reduced overall and progression-free survival [14]. Yamamoto et al. further reported that PDCs were associated with both reduced progression-free and overall survival, and that TB was also associated with reduced PFS [13]. The present study supports the findings that, in addition to TB, PDCs are also associated with reduced overall and progression-free survival. Ocal and Guzelis, when evaluating the prognostic impact of TB separately in grade I, pT1, or stage I endometrial carcinomas, also demonstrated an association between TB and reduced overall survival [15]. In the present study, TB and PDCs were associated with reduced overall and progression-free survival, particularly in low-grade cases. In the study by Rau et al., TB was highlighted as an independent prognostic determinant of overall and recurrence-free survival, particularly in early-stage tumors [12]. However, consistent with the present findings, the role of TB in advanced-stage tumors requires further investigation due to limited case numbers.

As in previous studies, we did not find a significant association between the MELF pattern and prognosis [12,13]. In the present study, TB and PDCs provided more robust prognostic information than the MELF pattern, consistent with observations reported in the literature. The findings suggest that the presence of TB and PDCs may represent significant determinants of prognosis in EEC. This observation is particularly noteworthy given the limited data in the literature regarding the relationship between PDCs and prognosis. The molecular mechanisms underlying PDCs and TB have not been sufficiently investigated; however, both have been identified as prognostic factors, and their combined assessment in EEC may enhance prognostic discrimination, as observed in colorectal cancer [18].

Rau and colleagues demonstrated that, when endometrial carcinoma cases were classified into molecular subgroups, the most pronounced effects in regression analyses were observed in the no specific molecular profile (NSMP) subgroup and in MMR deficiency (MMRd) cases, in which TB demonstrated a significant prognostic impact [12]. Stögbauer et al. also demonstrated that TB is an independent prognostic factor in microsatellite instability (MSI)–associated EEC. They reported that TB, defined as three or more buds per one high-power field, was associated with poor outcomes and LNM [16]. Similarly, in the present study, the presence of TB at a cut-off value of three and PDCs at a cut-off value of one in MMRd cases was associated with reduced overall survival, and both thresholds were also associated with LVI and LNM. The evaluation of TB and PDCs may assist in identifying MMRd patients with poorer prognosis who might benefit from procedures such as lymphadenectomy. Although TB has not yet been validated as a preselective marker for a specific molecular subtype, Rau et al. reported fewer tumor buds in POLE-mutated cases and higher TB counts in p53-abnormal cases [12]. Therefore, TB may prove useful in predicting POLE mutations in settings where molecular testing is not universally accessible, potentially contributing to the avoidance of overtreatment in high-grade tumors. Although molecular subtyping was not the primary focus of this study, the prognostic impact of TB and PDCs may also be interpreted within the framework of The Cancer Genome Atlas and European Society of Gynaecological Oncology molecular classification systems. In particular, the majority of EEC cases fall into the no specific molecular profile (NSMP) subgroup, in which robust histopathological risk stratifiers remain clinically relevant. In this context, TB and PDCs may provide additional prognostic information beyond molecular status, particularly in MMRp or NSMP tumors. Conversely, in molecularly defined high-risk groups such as p53-abnormal carcinomas, aggressive behavior is largely driven by underlying molecular alterations, which may limit the incremental prognostic value of TB and PDCs. These observations support the complementary role of TB and PDCs as morphology-based prognostic markers within an integrated molecular–histopathological risk assessment approach.

The present study has several limitations. Formal interobserver agreement analysis for TB and PDCs assessment was not performed. However, all evaluations were conducted independently by three pathologists using standardized and predefined criteria, with consensus review in discrepant cases, which may have reduced observer-related variability. Another limitation relates to the use of multiple cut-off values for TB and PDCs. Although these thresholds were prespecified based on previously published studies, the evaluation of more than one cut-off may increase the risk of multiplicity. In addition, the number of progression events was relatively limited, which may have affected the statistical power of the survival analyses, particularly in subgroup evaluations. Therefore, the present findings should be interpreted with caution and warrant validation in larger, independent cohorts. Finally, microsatellite instability (MSI) testing by polymerase chain reaction or next-generation sequencing was not performed; thus, MSI status was inferred solely from MMR immunohistochemistry. While MMR IHC is widely accepted as a reliable surrogate for MSI status in endometrial carcinoma, the absence of direct molecular MSI testing represents an inherent limitation of the study.

In conclusion, the presence of TB and PDCs in endometrial carcinoma represents an easily applicable and reproducible histopathological parameter. As demonstrated in the present study, both TB and PDCs were significantly associated with overall and progression-free survival, as well as with adverse clinicopathological features. These findings suggest that TB and PDCs may provide additional prognostic information and potentially contribute to treatment stratification. However, these results should be interpreted in light of the retrospective design of the study and the lack of universal standardization in TB and PDCs assessment. Therefore, while TB and PDCs—readily assessable without the need for additional immunohistochemical markers—appear to be promising candidates for routine reporting alongside the MELF pattern in EEC, their widespread implementation should ideally be supported by further prospective, standardized studies.

## Supplementary materials

**Table S1:** Overall survival analyses according to tumor budding (TB) and poorly differentiated clusters (PDC) using Kaplan–Meier and Cox proportional hazards models.

Variable	Cut-off	High-risk (≥) group n (events)	Reference group (<) n (events)	HR	95% CI	p-value (log-rank)
Tumor budding (TB)	≥1 vs <1	67 (20)	123 (16)	2.94	1.50–5.76	0.001
	≥3 vs <3	33 (12)	157 (24)	3.47	1.72–7.00	<0.001
Poorly differentiated cluster (PDC)	≥1 vs <1	66 (20)	124 (16)	3.24	1.66–6.32	<0.001
	≥3 vs <3	15 (6)	175 (30)	4.98	2.03–12.17	<0.001

Tumor budding (TB) and poorly differentiated clusters (PDCs) were analyzed using two predefined cut-off values (≥1 and ≥3). Overall survival (OS) was evaluated by Kaplan–Meier analysis with log-rank testing, and hazard ratios (HRs) with 95% confidence intervals (CIs) were estimated using univariable Cox proportional hazards regression models. Event numbers represent death from any cause. Percentages and event counts are shown for each subgroup.

Abbreviations: TB, tumor budding; PDCs, poorly differentiated clusters; HR, hazard ratio; CI, confidence interval.

**Table S2:** Cox proportional hazards analysis of tumor budding (TB) and poorly differentiated clusters (PDCs) for overall survival.

Variable	HR (Exp(B))	95% CI	p-value	Model
Tumor budding (ordinal: 0 / 1–2 / ≥3)	2.05	1.40–3.00	<0.001	Univariable Cox
Poorly differentiated clusters (ordinal: 0 / 1–2 / ≥3)	2.66	1.68–4.21	<0.001	Univariable Cox

This table summarizes the Cox proportional hazards analyses evaluating the prognostic impact of tumor budding (TB) and poorly differentiated clusters (PDCs) burden on overall survival. Kaplan–Meier analyses demonstrated progressively worse survival with increasing TB categories (no TB: n = 123, events=16; low TB [1–2]: n = 34, events=8; high TB [≥3]: n = 33, events = 12) and PDC categories (PDCs 0: n = 124, events=16; low PDCs [1–2]: n = 51, events=14; high PDC [≥3]: n = 15, events=6). Consistently, Cox regression confirmed a burden-dependent increase in mortality risk.

**Table S3:** Subgroup analyses of overall survival according to tumor budding (TB) and poorly differentiated clusters (PDCs).

Subgroup	Tumor budding /PDCs	Cut-off	High-risk (≥) group n (events)	Reference group (<) n (events)	HR	95% CI	p-value log-rank
Low-grade tumors	Tumor budding	≥3 vs <3	25 (7)	149 (21)	3.00	1.26–7.14	0.009
	PDCs	≥1 vs <1	52 (12)	122 (16)	2.28	1.07–4.86	0.029
MMRd tumors	Tumor budding	≥3 vs <3	10 (5)	43 (5)	12.28	2.39–63.09	<0.001
	PDCs	≥1 vs <1	25 (8)	28 (2)	8.51	1.67–43.29	0.003

Overall survival (OS) was analyzed using the Kaplan–Meier method and compared by log-rank test. Hazard ratios (HRs) and 95% confidence intervals (CIs) were calculated using univariable Cox proportional hazards regression models. Tumor budding (TB) and poorly differentiated clusters (PDCs) were evaluated as binary variables using the indicated cut-off values. Subgroup analyses were performed in low-grade tumors and mismatch repair–deficient (MMRd) tumors.

Abbreviations: TB, tumor budding; PDC, poorly differentiated clusters; MMRd, mismatch repair–deficient; HR, hazard ratio; CI, confidence interval.

## Figures and Tables

**Table 1:** Clinicopathological characteristics of the study cohort.

Features	Number of cases (%) or mean (range)	Features	Number of cases (%) or mean (range)

Age of surgery (n = 190)	59 (30–82)	Tumor size	
		- <4 cm	87 (45.8)
		- ≥ 4 cm	103 (54.2)

FIGO Grade			
- 1	80 (42.1)	Myometrial invasion	
- 2	94 (49.5)	- <1/2	82 (43.2)
- 3	16 (8.4)	- ≥1/2	108 (56.8)

Cervical stromal involvement		Lymphovascular invasion	
- Present	40 (21,1)	- Present	55 (28.9)
- Absent	150 (78.9)	- Absent	135 (71.1)

Lymph node metastasis		Serosal involvement	
- Present	27 (14,2)	- Present	31 (16.3)
- Absent	163 (85.8)	- Absent	159 (83.7)

Pattern of myometrial invasion			
- Pushing pattern	70 (36.8)	MELF pattern	
- Infiltrative	39 (20.5)	- Present	58 (30.5)
- MELF	58 (30.5)	- Absent	132 (69.5)
- Adenomyosis-like	23 (12.1)		

Tumor budding		Poorly differentiated clusters	
“Cut off 1”		“Cut off 1”	
- ≥1	67 (35.3)	- ≥1	66 (34.7)
- <1	123 (64.7)	- <1	124 (65.3)
“Cut off 3”		“Cut off 3”	
- ≥3	33 (17.4)	- ≥3	15 (7.9)
- <3	157 (82.6)	- <3	175 (92.1)

- MELF (−), TB (+)	27 (14.2)	MMR proteins	
- MELF (+), TB (−)	18 (9.5)	- MMRp	117 (61.6)
- MELF (+), TB (+)	40 (21.1)	- MMRd	53 (27.9)
- MELF (−), TB (−)	105 (55.3)	- Unknown	20 (10.5)

FIGO Stage		pT	
- Early (I–II)	153 (80.5)	- T1–T2	152 (80)
- Advanced (III–IV)	37 (19.5)	- ≥ T3	38 (20)

Survival (month)	68	Mortality	
		- Alive	154 (81.1)
		- Deceased	36 (18.9)

MELF: microcystic, elongated, and fragmented pattern; TB: tumor budding; MMR: mismatch repair; MMRd: mismatch repair–deficient; MMRp: mismatch repair–proficient.

**Table 2:** Associations between the MELF pattern, tumor budding (TB), and poorly differentiated clusters (PDCs) and clinicopathological parameters according to different cut-off values.

	Tumor budding (TB)						Poorly differentiated clusters (PDCs)						MELF		

	1 cut-off point			3 cut-off point			1 cut-off point			3 cut-off point					

Parameters	≥1 (n = 67)	<1 (n = 123)	p value	≥3 (n = 33)	<3 (n = 157)	p value	≥1 (n = 66)	<1 (n = 124)	p value	≥3 (n = 15)	<3 (n = 175)	p value	Present (n = 58)	Absent (n = 132)	p value

**Tumor diameter**															
**<4 cm (n = 87)**	24 (27.6%)	63 (72.4%)	**0.042**	9 (10.3%)	78 (89.7%)	**0.019**	23 (26%)	64 (74%)	**0.027**	5 (5.7%)	82 (94.3%)	0.313	16 (18%)	71(82%)	**0.001**
**≥ 4 cm (n = 103)**	43 (41.7%)	60 (58.3%)		24 (23.3%)	79 (76.7%)		43 (42%)	60 (58%)		10 (9.7%)	93 (90.3%)		42 (41%)	61 (59%)	

**FIGO grade**															
**Low (1–2) (n = 174)**	53 (30.5%)	121 (69.5%)	**<0.001**	25 (14.4%)	149 (85.6%)	**<0.001**	52 (30%)	122 (70%)	**<0.001**	7 (4%)	167 (96%)	**<0.001**	56 (32%)	118 (68%)	0.102
**High (3) (n = 16)**	14 (87.5%)	2 (12.5%)		8 (50%)	8 (50%)		14 (87.5%)	2 (12.5%)		8 (50%)	8 (50%)		2 (12.5%)	14 (87.5%)	

**Myometrial invasion**															
**<1/2 (n = 82)**	8 (9.8%)	74 (90.2%)	**<0.001**	4 (4.9%)	78 (95.1%)	**<0.001**	8 (10%)	74 (90%)	**<0.001**	2 (2.4%)	80 (97.6%)	**0.015**	10 (12%)	72 (88%)	**<0.001**
**≥1/2 (n = 108)**	59 (54.6%)	49 (45.4%)		29 (26.8%)	79 (73.2%)		58 (54%)	50 (46%)		13 (12%)	95 (88%)		48 (44%)	60 (56%)	

**Cervical stromal involvement**															
**Present (n = 40)**	25 (62.5%)	15 (37.5%)	**<0.001**	14 (35%)	26 (65%)	**<0.001**	23 (57.5%)	17 (42.5%)	**0.001**	5 (12.5%)	35 (87.5%)	0.224	20 (50%)	20 (50%)	**0.003**
**Absent (n = 150)**	42 (28%)	108 (72%)		19 (12.7%)	131 (87.3%)		43 (29%)	107 (71%)		10 (6.7%)	140 (93.3%)		38 (25%)	112 (75%)	

**Lymphovascular invasion**															
**Present (n = 55)**	47 (85.5%)	8 (14.5%)	**<0.001**	29 (52.7%)	26 (47.3%)	**<0.001**	47 (85%)	8 (15%)	**<0.001**	13 (24%)	42 (76%)	**<0.001**	31 (56%)	24 (44%)	**<0.001**
**Absent (n = 135)**	20 (14.8%)	115 (85.2%)		4 (3%)	131 (97%)		19 (14%)	116 (86%)		2 (1.5%)	133 (98.5%)		27 (20%)	108 (80%)	

**Lymph node metastasis**															
**Present (n = 27)**	25 (92.6%)	2 (7.4%)	**<0.001**	16 (59.3%)	11 (40.7%)	**<0.001**	24 (89%)	3 (11%)	**<0.001**	6 (22%)	21 (78%)	**0.003**	20 (74%)	7 (26%)	**<0.001**
**Absent (n = 163)**	42 (25.7%)	121 (74.3%)		17 (10.4%)	146 (89.6%)		42 (26%)	121 (74%)		9 (5.5%)	154 (95.5%)		38 (23%)	125 (77%)	

**Serosal involvement**															
**Present (n = 31)**	28 (90.3%)	3 (9.7%)	**<0.001**	22 (71%)	9 (29%)	**<0.001**	27 (87%)	4 (13%)	**<0.001**	11 (35.5%)	20 (64.5%)	**<0.001**	18 (58%)	13 (42%)	**<0.001**
**Absent (n = 159)**	39 (24.5%)	120 (75.5%)		11 (7%)	148 (93%)		39 (25%)	120 (75%)		4 (2.5%)	155 (97.5%)		40 (25%)	119 (75%)	

**FIGO Stage**															
**Low (stage 1–2) (n = 153)**	34 (22.2%)	119 (77.8%)	**<0.001**	13 (8.5%)	140 (91.5%)	**<0.001**	35 (23%)	118 (77%)	**<0.001**	6 (4%)	147 (96%)	**<0.001**	35 (23%)	118 (77%)	**<0.001**
**High (stage 3–4) (n = 37)**	33 (89.2%)	4 (10.8%)		20 (54%)	17 (46%)		31 (84%)	6 (16%)		9 (24%)	28 (76%)		23 (62%)	14 (38%)	

**T stage**															
**Early (T1–T2) (n = 152)**	33 (21.7%)	119 (78.3%)	**<0.001**	12 (7.9%)	140 (92.1%)	**<0.001**	34 (22%)	118 (78%)	**<0.001**	6 (4%)	146 (96%)	**<0.001**	34 (22%)	118 (78%)	**<0.001**
**Advanced (≥T3) (n = 38 )**	34 (89.5%)	4 (10.5%)		21 (55.3%)	17 (44.7%)		32 (84%)	6 (16%)		9 (24%)	29 (76%)		24 (63%)	14 (37%)	

**Distant metastasis**															
**Present (n = 11)**	9 (81.8%)	2 (18.2%)	**<0.001**	4 (36.4%)	7 (63.6%)	0.087	9 (82%)	2 (18%)	**0.001**	2 (18%)	9 (82%)	0.192	3 (27%)	8 (73%)	0.809
**Absent (n = 179)**	58 (32.4%)	121 (67.6%)		29 (16.2%)	150 (83.8%)		57 (32%)	122 (68%)		13 (7.3%)	166 (92.7%)		55 (31%)	124 (69%)	

* **MMR proteins (n = 170)**															
**MMRd (n = 53)**	26 (49%)	27 (51%)	**0.006**	10 (19%)	43 (81%)	0.571	25 (47%)	28 (53%)	**0.016**	3 (5.7%)	50 (94.3%)	0.632	24 (45%)	29 (55%)	**0.011**
**MMRp (n = 117)**	32 (27.4%)	85 (72.6%)		18 (15%)	99 (85%)		33 (28%)	84 (72%)		9 (8%)	108 (92%)		30 (26%)	87 (74%)	

**MELF pattern**															
**Present (n = 58)**	40 (69%)	18 (31%)	**<0.001**	22 (38%)	36 (62%)	**<0.001**	40 (69%)	18 (31%)	**<0.001**	7 (12%)	51 (88%)	0.157			
**Absent (n = 132)**	27 (20.4%)	105 (79.6%)		11 (8%)	121 (92%)		26 (20%)	106 (80%)		8 (6%)	124 (94%)				

Percentages were calculated within each respective subgroup. Tumor budding (TB) and poorly differentiated clusters (PDCs) were evaluated using predefined cut-off values of one and three.

* Cases with unknown MMR protein expression (n = 20) were excluded only from the MMR subsection of this table; all other analyses included the full cohort where applicable.

Bold values indicate statistically significant results (p < 0.05).

**Table 3:** Univariate and multivariable analyses of lymphovascular invasion (LVI) and lymph node metastasis (LNM) in endometrioid endometrial carcinoma (EEC).

Univariate analyses				
Clinicopathological parameters	LVI		LNM	
	p value		p value	
FIGO grade	**<0.001**		**0.041**	
Tumor size <4 vs. ≥4	**<0.001**		**0.008**	
Depth of invasion <half vs. ≥half	**<0.001**		**<0.001**	
Cervical involvement + vs. −	**<0.001**		**<0.001**	
Serosal involvement + vs. −	**<0.001**		**<0.001**	
MELF pattern + vs. −	**<0.001**		**<0.001**	
Tumor budding (≥1) ≥1 vs. <1	**<0.001**		**<0.001**	
PDC (≥1) ≥1 vs. <1	**<0.001**		**<0.001**	
LVI + vs. −			**<0.001**	
LNM + vs. −	**<0.001**			
**Multivariable analyses**				
**Clinicopathological parameters**	**LNM**			
	Model	OR	95% CI	p-value
Tumor budding (≥1) ≥1 vs. <1		11.14	1.43–86.44	**0.021**
MELF pattern + vs. −		3.26	1.15–9.28	**0.027**
PDCs (≥1) ≥1 vs. <1		2.39	0.40–14.42	0.342
**Clinicopathological parameters**	**LVI**			
	Model A	OR	95% CI	**p-value**
Tumor budding (≥1) ≥1 vs. <1		28.73	11.11–74.26	**<0.001**
MELF pattern + vs. −		1.49	0.60–3.71	0.395
	Model B	OR	95% CI	**p-value**
PDCs (≥1) ≥1 vs. <1		31.34	11.93–82.36	**<0.001**
MELF pattern + vs. −		1.37	0.54–3.49	0.507

Model A and Model B were constructed to avoid collinearity between tumor budding (TB) and poorly differentiated clusters (PDCs). The LNM model included 27 events, whereas the LVI models included 55 events. Variables were selected a priori based on clinical relevance and univariate significance in order to maintain an adequate events-per-variable ratio. OR: odds ratio; CI: confidence interval; TB: tumor budding; PDCs: poorly differentiated clusters; MELF: microcystic, elongated, and fragmented pattern. (Multivariable analyses: LVI Model A, Nagelkerke R_2_ = 0.53; LVI Model B, Nagelkerke R_2_ = 0.54; LNM model, Nagelkerke R_2_ = 0.44). Bold values indicate statistically significant results (p < 0.05).
